# Supplementary material for: Chemical Intolerance Is Associated with Autism Spectrum and Attention Deficit Disorders: A Five-Country Cross-National Replication Analysis
Source: J Xenobiot. 2026 Jan 1;16(1):5. doi: 10.3390/jox16010005 (PMC12821635; doi:10.3390/jox16010005)
Supplement: Supplementary file 1 [file jox-16-00005-s001.zip › jox-3983625-supplementary.pdf]

Journal of Xenobiotics

MDPI

Supplementary Materials: Chemical Intolerance Is Associated with Autism Spectrum and Attention Deficit Disorders: A Five-Country Cross-National Replication Analysis

Raymond Palmer and David Kattari

Dynata – ASD by Age Category and Country

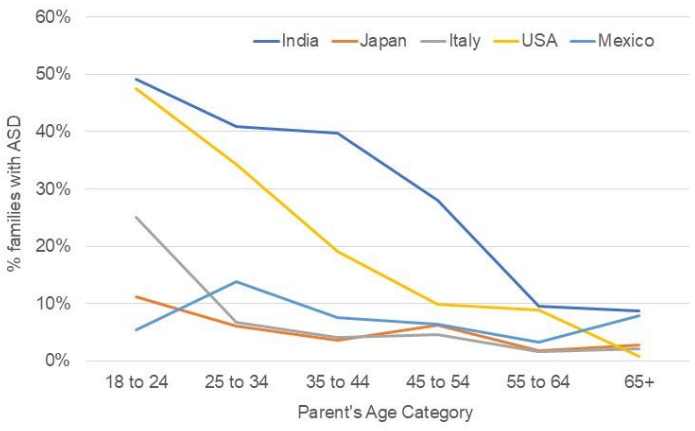

Figure S1. Maternal self-reported ASD by age category and country

Dynata – ADHD by Age Category and Country

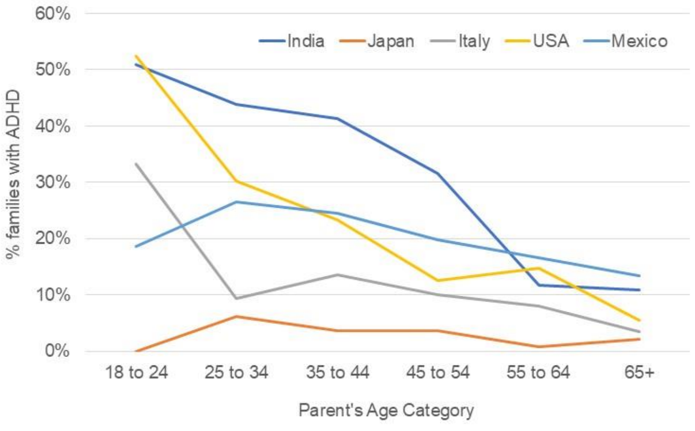

Figure S2. Maternal self-reported ADHD by age category and country.

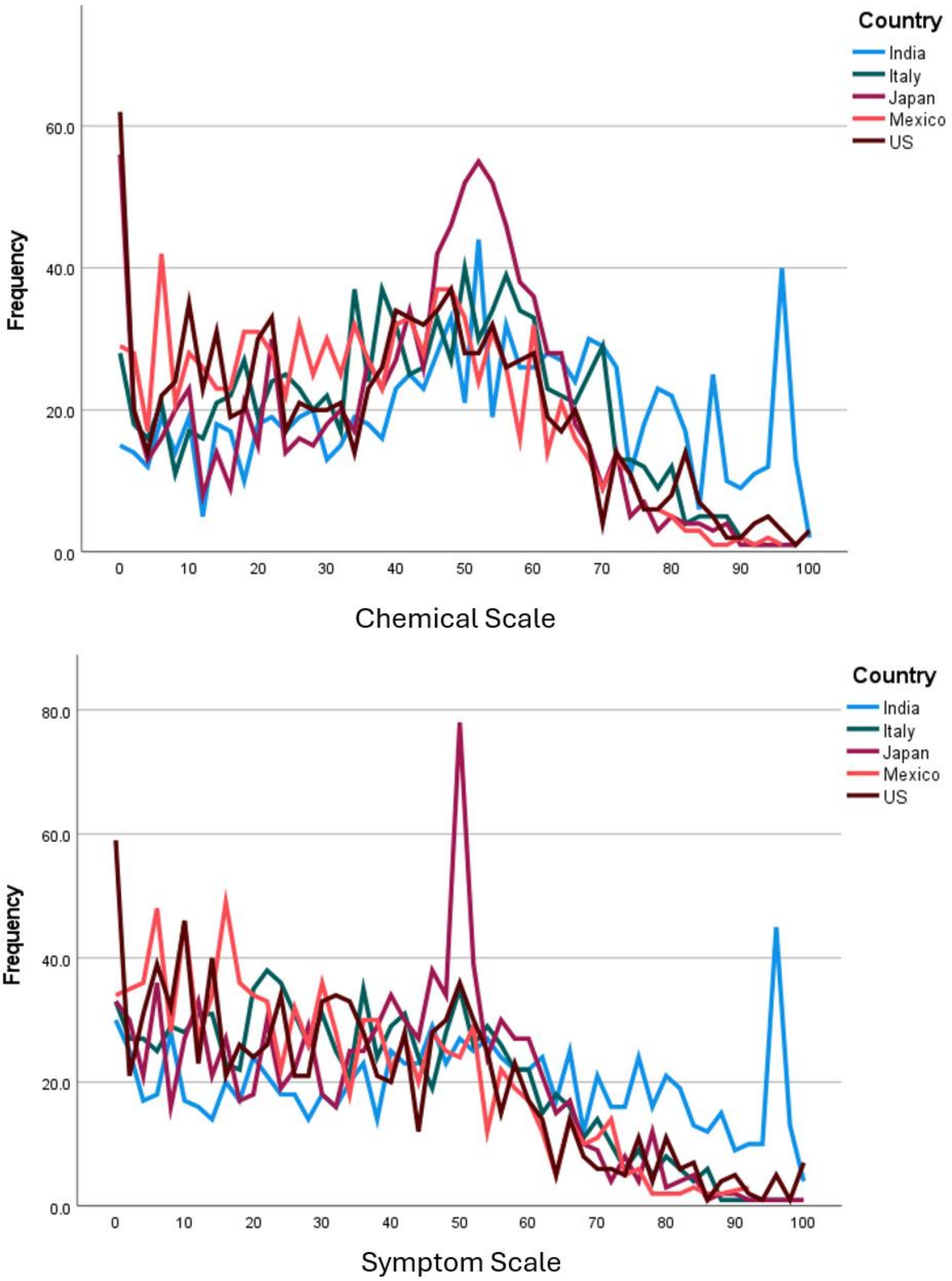

Figure S3. Frequency of responses on the QEESI Chemical and Symptom Scales by country

Note: Japan's response tendency for central responses on Likert-type scales such as these is consistent with other research [61].
